# Association of smoking status with prognosis in bladder cancer: A meta-analysis

**DOI:** 10.18632/oncotarget.13606

**Published:** 2016-11-25

**Authors:** Lina Hou, Xuwei Hong, Meng Dai, Pengliang Chen, Hongfan Zhao, Qiang Wei, Fei Li, Wanlong Tan

**Affiliations:** ^1^ Department of Healthy Management, Nanfang Hospital, Southern Medical University, Guangzhou, Guangdong 510515, P. R. China; ^2^ Department of Urology, Nanfang Hospital, Southern Medical University, Guangzhou, Guangdong 510515, P.R.China

**Keywords:** smoking, bladder cancer, prognosis, surgery, meta-analysis

## Abstract

There is considerable controversy regarding the association between smoking and prognosis in surgically treated bladder cancer. The present meta-analysis was performed to quantify the role of smoking status in bladder cancer recurrence, progression and patient survival by pooling the available previous data. Pubmed, Embase and the Cochrane Library databases were searched for eligible studies published prior to April 2016. Random and fixed effects models were used to calculate the summary relative risk estimates (SRRE). A total of 10,192 patients from 15 studies were included in the meta-analysis. There was evidence of positive associations between current smoking and the risk of recurrence (SRRE=1.23; 95% CI, 1.05–1.45) and mortality (SRRE=1.28; 95% CI, 1.07-1.52) in bladder cancer. Furthermore, former smoking had positive associations with bladder cancer recurrence (SRRE=1.22; 95% CI, 1.09-1.37) and mortality (SRRE=1.20; 95% CI, 1.03-1.41). However, there was no significant association between bladder cancer progression risk and current (SRRE=1.11; 95% CI, 0.71-1.75) or previous smoking (SRRE=1.16; 95% CI, 0.92-1.46). The findings indicate that current and former smoking increase the risk of recurrence and mortality in patients with bladder cancer. However, due to the nonrandomized and retrospective nature of the current study, patients may be prone to potential selection bias. Prospective and larger epidemiological studies with a longer follow-up are required to confirm these findings.

## INTRODUCTION

Bladder cancer is the most frequently diagnosed cancer in the urinary tract and is the ninth most common cancer worldwide [1]. Approximately 75% of newly diagnosed bladder cancers are noninvasive, and this type has a high rate of recurrence and progression despite the use of local therapy. The probability of recurrence at 5 years ranges from 31-78% and the probability of progression ranges from 1-45% [2]. The remaining 25% of bladder cancer cases (invasive) either require radical surgery or systemic therapy, including chemotherapy. However patient outcome often remains poor despite systemic therapy. Following radical cystectomy (RC), the 5-year recurrence-free survival rate is 58-65%, and the cancer-specific survival is 60-66% [3, 4]. Thus, it is important to investigate what factors affect the prognosis of bladder cancer patients following surgery.

Tobacco smoking is a well-established risk factor for bladder cancer development, acting to increase the risk by 2- to 4-fold. In most populations, smoking is related to more than half of bladder cancer cases in men and a third of cases in women [5]. In the past several decades, smoking has gradually declined [6]. Smoking status and the effect on the outcome of bladder cancer after surgery has gained considerable research attention over the last few years; however, the analyses have yielded somewhat inconsistent results. Some reports indicated that quitting smoking reduced the risk of disease recurrence, disease progression and cancer-specific mortality [7–9], whereas other studies detected no significant association between smoking status and the prognosis of patients with bladder cancer [10–12]. A recent systematic review examined the relationship between smoking status and outcome of urothelial carcinoma patients; however, no analysis was performed using data pooled from multiple previous studies [13].

Evidence of the prognostic significance of smoking status may emphasize the importance of urologists in improving patient awareness of the risks of smoking and the benefits of quitting. Furthermore, it may highlight the importance of determining the smoking status of patients when making clinical treatment decisions. The aim of this study was to examine the association of smoking status with prognosis in bladder cancer patients following surgery, and to explore potential sources of heterogeneity across previous studies by performing a meta-analysis.

## RESULTS

### Literature search results

**Figure 1:**
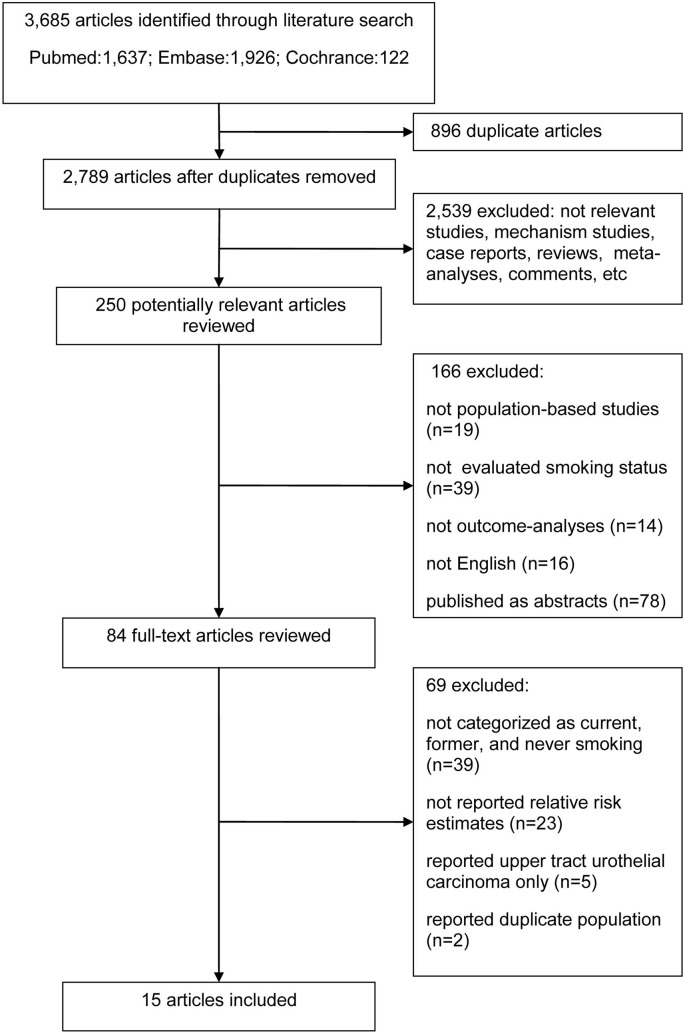
Flow diagram of study selection

Our search strategy identified 3,685 articles, of which 250 were considered to be potentially relevant articles following the exclusion of irrelevant studies, including mechanistic studies, case reports, reviews, meta-analyses, meeting abstracts and comments. Of these, 166 articles were excluded because the studies were not population-based studies, the smoking status was not evaluated or the related outcomes were not reported in the analyses. The remaining 84 studies were assessed by full text review, and 39 articles were excluded because the smoking status was not categorized as current smoker, former smoker and never smoker in these studies. Additionally, 23 studies did not report the relative risk estimates and the corresponding 95% CI, or did not provide sufficient information to estimate them. Furthermore, the patients in another 5 studies had upper tract urothelial carcinoma treated with radical nephroureterectomy, and these 5 studies were therefore excluded. In addition, 2 studies were excluded due to an overlap of the study population with that of another study [14, 15]. Therefore, a total of 15 studies published between 1995 and 2015 were finally included in our meta-analysis. A flow chart of the search and selection process is shown in Figure 1.

### Study characteristics

The characteristics of the studies included in the meta-analysis are presented in Table 1. A total of 10,192 patients from 13 cohort studies and 2 case-control studies were pooled to examine the relationship between smoking status and patient prognosis in bladder cancer treated with surgery. Of these 15 studies, 3 were conducted in multi-national centers [7, 8, 16], 4 in the United States [17–20], 2 in Italy [11, 21], and 1 in each of the Netherlands [10], Canada [22], China [9], Korea [12], India [23] and Lebanon [24]. In 10 studies, 6,307 patients were treated with transurethral resection of the bladder (TURB), and the 3,885 patients in the other 5 reports underwent RC. The outcomes identified were disease recurrence in 15 studies, disease progression in 5 studies and cancer-specific mortality in 5 studies. The Newcastle-Ottawa scale (NOS) score of the included studies ranged from 7 to 9, indicating that all the studies were of high quality (Table 2).

**Table 1:** Characteristics of studies included in meta-analysis of smoking status and bladder cancer outcomes

Study	Setting (Country)	Period	Mean follow-up (months)	Sample size	Cur: For: Non	Mean age (years)	Sex (%)	Disease stage	NOS score	Outcomes of Cur vs. Non and For vs. Non (SRRE 95%CI)		
										Recurrence	Progression	Cancer-specific mortality
Pastore 2015 [21]	Database archive of the Department of Urology (Italy)	2008~2003	45.1	574	200: 216: 158	62.2	male 100	NMIBC	8	3.20(1.98-5.17)^a^ 2.19(1.38-3.48)^a^		
Grotenhuis 2015 [10]	The Netherlands Cancer Registry (Netherlands)	1995~2010	44.4	963	292: 490: 181	63.9	male 82.1; female 17.9	NMIBC	8	0.93(0.67-1.29); 1.14(0.85-1.53)	0.80(0.45-1.42); 1.36(0.84-2.21)	
Wyszynski 2014 [24]	The New Hampshire State Cancer Registry (Lebanon)	1994~2001	72.0	726	214: 379: 123	61.6	male 76.4; female 23.6	NMIBC	8	1.51(1.08-2.13); 1.61(1.17-2.20)		
Kim 2014 [17]	Memorial Sloan-Kettering Cancer Center (USA)	1990~2011	46.0	139	41: 63: 35	65.0	male 71.2, female 28.8	MIBC	8	0.91(0.44-1.84); 1.24(0.66-2.31)		1.07(0.44-2.60); 0.90(0.40-2.03)
Serretta 2013 [11]	A randomized multicenter trial (Italy)	2002~2003	48.0	395	298: 97^c^	68.0	Male 86.1, female 13.9	NMIBC	9	1.39(0.40-2.24); 1.94(1.18-3.18)		
Rink 2013 [7]	Six international centers	1987~2007	49.0	2043	593: 956: 494	67.0	Male 78.7, female 21.3	NMIBC	8	1.22(1.01-1.48); 1.12(0.94-1.34)	2.09(1.29-3.39); 1.29(0.79-2.09)	1.12(0.85-1.47); 1.10(0.86-1.41)
Rink 2013 [8]	Five international centers	2000~2008	34.3	1506	517: 693: 296	66.4	Male 77.3, female 22.7	MIBC	8	1.47(1.12-1.94); 1.26(0.96-1.66)		1.41(1.04-1.90); 1.22(0.91-1.63)
Da Silva 2013 [16]	Four international institutions	1992~2008	34.0	1502	516: 693: 293	66.0	Male 78.4, female 21.6	MIBC	8	1.47(1.12-1.92); 1.27(0.98-1.65)		1.43(1.06-1.93); 1.24(0.98-1.66)
Lee 2012 [12]	The Asan Medical Center (Korea)	1989~2008	56.0	602	159: 181: 262	62.2	Male 89.7, female 10.3	Both	8	0.91(0.63-1.31); 0.93(0.66-1.29)		0.94(0.64-1.37); 1.21(0.86-1.70)
Sfakianos 2011 [18]	The Memorial Sloan-Kettering Cancer Center (USA)	1994~2008	80.9	623	97: 386: 140	75.0	Male 67.9, female 32.1	NMIBC	7	1.04(0.77-1.40)^b^ 1.05(0.84-1.32)^b^	1.16(0.65-2.10)^b^ 1.00(0.64-1.58)^b^	1.27(0.64-2.53)^b^ 1.14(0.66-1.97)^b^
Chade 2010 [19]	The Memorial Sloan-Kettering Cancer Center (USA)	1998~2008	48.0	155	11: 91: 44	69.0	Male 85.2; female 14.8	NMIBC	7	1.22(0.46-3.21)^b^ 1.16(0.62-2.18)^b^ .	0.86(0.35-2.11)^b^ 1.02(0.63-1.66)^b^	
Ahirwar 2008 [23]	Sanjay Gandhi Postgraduate Institute of Medical Sciences (India)	2004~2007	13.0	136	46: 27: 63	61.6	Male 87.5; female 12.5	Both	8	0.72(0.37-2.16); 1.17(0.59-2.31)		
Chen 2007 [9]	National Taiwan university hospital (China)	1997~2005	38.0	265	78: 123: 64	67.1	male 100	NMIBC	9	1.00(0.55-1.82); 0.64(0.32-1.23)		
Leibovici 2005 [20]	Anderson Cancer Center & Baylor College of Medicine (USA)	1995~2003	20.8	195	44: 99: 52	62.6	Male 76.9; female 23.1	NMIBC	7	0.81(0.47-1.37); 1.11(0.73-1.70)	0.59(0.17-2.03); 1.30(0.53-3.16)	
Allard 1995 [22]	Fifteen acute-care participating hospitals (Canada)	1990~1992	23.7	368	162: 150: 56	65.1	Male 73.6; female 26.4	NMIBC	7	1.45(0.94-2.24); 1.28(0.82-1.98)		

Abbreviations: Cur, current smokers; For, former smokers; Non, non-smokers; SRRE, summary relative risk estimates; CI, confidence interval; NOS, Newcastle-Ottawa scale; MIBC, muscle invasive bladder cancer; NMIBC, non-muscle invasive bladder cancer.

a the effect estimates in this study is odd ratio (OR) with 95%CI;

b the HR and 95%CI is extracted from the univariate model;

c only reported the sample size of smoker and non-smoker.

**Table 2:** Assessment of quality of studies by Newcastle-Ottawa Scale

Studies	Selection			Comparability			Outcome			Total
	1	2	3	4	5A	5B	6	7	8	
Pastore 2015[21]	*	*		*	*	*	*	*	*	8
Grotenhuis 2015[10]	*	*		*	*	*	*	*	*	8
Wyszynski 2014[24]	*	*		*	*	*	*	*	*	8
Kim 2014[17]	*	*		*	*	*	*	*	*	8
Serretta 2013[11]	*	*	*	*	*	*	*	*	*	9
Rink 2013[7]	*	*		*	*	*	*	*	*	8
Rink 2013[8]	*	*		*	*	*	*	*	*	8
Da Silva 2013[16]	*	*		*	*	*	*	*	*	8
Lee 2012[12]	*	*		*	*	*	*	*	*	8
Sfakianos 2011[18]	*	*	*	*			*	*	*	7
Chade 2010[19]	*	*			*	*	*	*	*	7
Ahirwar 2008[23]	*	*	*	*	*		*	*	*	8
Chen 2007[9]	*	*	*	*	*	*	*	*	*	9
Leibovici 2005[20]	*	*	*	*			*	*	*	7
Allard 1995[22]	*	*	*		*		*	*	*	7

1 = Representativeness of the exposed cohort;

2 = Selection of the non-exposed cohort;

3 = Ascertainment of exposure;

4 = Demonstration that outcome of interest was not present at start of study;

5 = Comparability of cohorts on the basis of the design or analysis (a. study controls for age; b. study controls for any additional factor);

6 = Assessment of outcome;

7 = Follow-up long enough for outcomes to occur;

8 = Adequacy of follow up of cohorts.

### Current smoking and prognosis in bladder cancer after surgery

**Figure 2:**
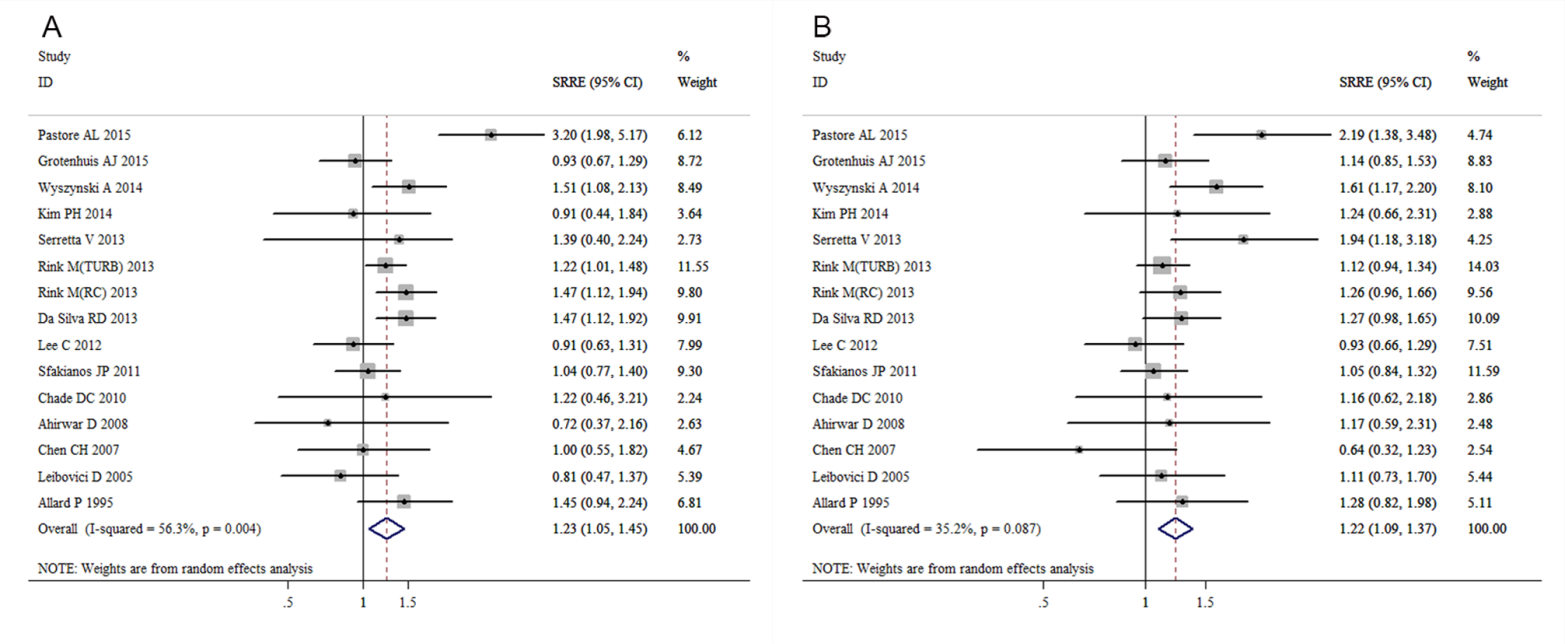
Meta-analysis of studies that examined the associations of bladder cancer recurrence risk with A. current and B. former smoking

**Figure 3:**
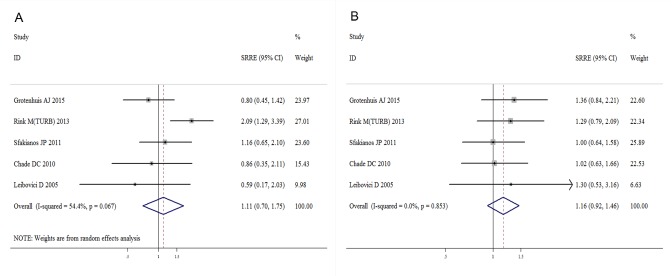
Meta-analysis of studies that examined the associations of bladder cancer progression risk with A. current and B. former smoking

**Figure 4:**
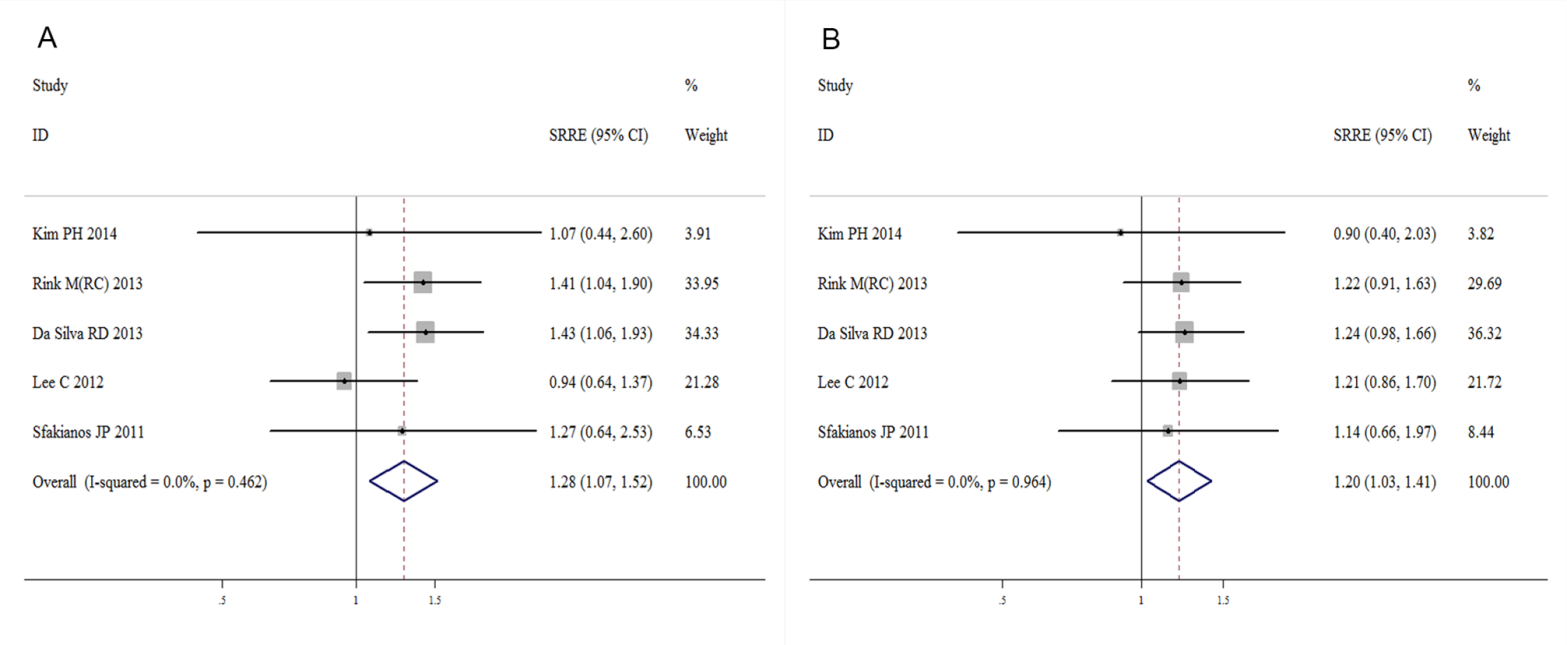
Meta-analysis of studies that examined the associations of bladder cancer mortality risk with A. current and B. former smoking

The meta-analysis revealed that current smoking was associated with an increased risk of bladder cancer recurrence compared with a never smoking status, with an SRRE of 1.23 (95% CI, 1.05-1.45). Substantial heterogeneity was observed across the studies (I^2^=56.3%; Q=32.02; heterogeneity P-value=0.004) (Figure 2A). No evidence of any association was detected between current smoking and disease progression (SRRE=1.11; 95% CI, 0.71-1.75) (Figure 3A). Significant variability was detected among the studies of disease progression (I^2^=54.4%; Q=8.77; heterogeneity P-value=0.067). Moreover, compared with never smoking, current smoking had a positive association with bladder cancer-specific mortality (SRRE=1.28; 95% CI, 1.07-1.52) (Figure 4A), and no substantial heterogeneity was indicated across the studies (I^2^=0.0%; Q=3.61, heterogeneity P-value=0.462). There was no statistical evidence of publication bias in the studies that measured disease recurrence (Begg, P=0.621; and Egger, P=0.697), disease progression (Begg, P=0.462; and Egger, P=0.217) and cancer-specific mortality (Begg, P=1.000; and Egger, P=0.413) in current smokers.

Subgroup and meta-regression analyses were conducted to explore the heterogeneity among studies assessing the relationship between current smoking and disease recurrence (Table 3). No statistically significant source of heterogeneity was identified. Sensitivity analyses were performed by sequentially excluding each study in turn to examine the influence of individual studies on the pooled results. The results indicated the significant association between current smoking and disease recurrence was reliable and robust (data not shown).

**Table 3:** Summary of meta-analysis results for smoking status and disease recurrence for bladder cancer

Analysis specification	n (size)	Current smoker vs. Never smoker				Former smoker vs. Never smoker			
		HR (95%CI)	I^2^(%)	*p-het*	*p-reg*	SRRE (95%CI)	I^2^(%)	*p-het*	*p-reg*
All	15 (10192)	1.23(1.05-1.45)	56.3	0.004		1.22(1.09-1.37)	35.2	0.087	
Study design					0.443				0.873
Single center	12 (5141)	1.17(0.94-1.47)	60.9	0.003		1.24(1.05-1.46)	46.8	0.037	
multicenter	3 (5051)	1.34(1.17-1.53)	0.0	0.403		1.19(1.04-1.35)	0.0	0.653	
Geographic region					0.910				0.335
America	5 (1480)	1.08(0.87-1.33)	0.0	0.522		1.11(0.94-1.31)	0.0	0.942	
Europe	3 (1932)	1.60(0.67-3.84)	88.5	0.000		1.64(1.05-2.55)	71.1	0.031	
Asia	4 (1729)	1.08(0.79-1.50)	43.5	0.151		1.08(0.73-1.60)	65.9	0.032	
Disease stage					0.356				0.409
MIBC	3 (3147)	1.42(1.18-1.71)	0.0	0.447		1.26(1.05-1.51)	0.0	0.997	
NMIBC	10 (6307)	1.27(1.02-1.57)	62.7	0.004		1.26(1.07-1.48)	52.7	0.025	
UBC	2 (738)	0.88(0.63-1.23)	0.0	0.631		0.97(0.72-1.31)	0.0	0.554	
Method of surgery					0.657				0.638
RC	5 (3885)	1.19(0.92-1.54)	48.9	0.098		1.18(1.01-1.38)	0.0	0.646	
TURB	10 (6307)	1.27(1.02-1.57)	62.7	0.004		1.26(1.07-1.48)	52.7	0.025	

Abbreviations: MIBC, muscle invasive bladder cancer; NMIBC, non-muscle invasive bladder cancer; TURB, Transurethral resection of the bladder; RC, Radical cystectomy; p-het, p value for heterogeneity; p-reg, p value for meta-regression; SRRE, summary relative risk estimates.

### Former smoking and prognosis in bladder cancer after surgery

A total of 15 studies assessed the association of former smoking with bladder cancer recurrence after surgery. A positive association with former smoking was observed among the studies that assessed disease recurrence (SRRE=1.22; 95% CI, 1.09-1.37), with evidence of heterogeneity detected (I^2^=35.2%; Q=21.59; heterogeneity P-value=0.087) (Figure 2B). Moreover, although there was no significant association between former smoking and disease progression (SRRE=1.16; 95% CI, 0.92-1.46) (Figure 3B), cancer-specific mortality was increased by 20% in former smokers compared with never smokers (SRRE=1.20; 95% CI, 1.03-1.41) (Figure 4B). There was no evidence of heterogeneity among the studies that recorded disease progression (I^2^=0.0%; Q=1.35; heterogeneity P-value=0.853) and cancer-specific mortality (I^2^=0.0%; Q=0.59; heterogeneity P-value=0.964) in former smokers. Furthermore, though no statistical evidence of publication bias was suggested by Begg's or Egger's tests in the meta-analyses of disease recurrence (Begg, P=0.806; and Egger, P=0.617) and disease progression (Begg, P=0.806; and Egger, P=0.617). However, the Begg's (P=0.027) and Egger's (P=0.013) tests of reports on cancer-specific mortality indicated that there was possible publication bias.

Among the subgroup analyses, although no significant modifiers that would cause inter-study heterogeneity were detected, the findings of subgroup analyses were consistently independent of study design, disease stage and surgical method (Table 3). Sensitivity analyses also suggested that no single study would influence the overall findings, which implied that our results were reliable and robust (data not shown).

## DISCUSSION

Cigarette smoking is thought to promote the development of bladder cancer; a higher incidence of bladder cancer is detected in smokers with a longer period of exposure to carcinogens (including polycyclic aromatic hydrocarbons, aromatic amines and nitrosamines) through cigarette smoking [25, 26]. However, studies investigating the efficacy of adjuvant treatments for the prevention of recurrence have rarely taken smoking status into consideration. Furthermore, epidemiological studies pay more attention to cancer incidence than to the outcome of bladder cancer following surgery. There is no comprehensive evidence on the association between smoking status and the prognosis of patients with bladder cancer after surgery.

To the best of our knowledge, this is the first systematic epidemiological assessment of the association between smoking status and the prognosis of patients with bladder cancer. A meta-analysis was conducted on 13 cohort studies and 2 case-control studies with 10,192 bladder cancer patients in total to provide a stable and credible result. The outcomes of bladder cancer treated with surgery included disease recurrence, disease progression and cancer-specific mortality. The summarized results indicated that current smoking may increase the risk of disease recurrence and cancer-specific mortality, but may not be associated with the risk of disease progression. Furthermore, former smoking was found to be positively associated with disease recurrence and cancer-specific mortality, but no significant association was detected between former smoking and disease progression.

Substantial heterogeneity was detected among the studies of current/former smoking and disease recurrence. Furthermore, some heterogeneity was observed among the studies that reported on current smoking and disease progression. The heterogeneity that existed in our analysis was potentially caused by differences in study populations, model selection for patients treated with surgery, variability of intravesical therapies, study design and the follow-up duration. Although subgroup analyses were performed to elucidate potential sources of heterogeneity, the source of heterogeneity was not identified. However, sensitivity analyses indicated that our findings were reliable and robust. In addition, there was no evidence of significant publication bias in these analyses according to the results of the Begg's or Egger's tests, except that the Begg's and Egger's tests indicated that there was possible publication bias in the studies that examined the association of former smoking with disease progression risk.

The overall findings indicated that current and former smokers were at a significantly higher risk of experiencing disease recurrence and cancer-specific mortality compared with patients that had never smoked. The biological mechanisms linking current and former smokers with recurrence and cancer-specific mortality are not well characterized. However, there are several mechanisms that are considered to have important roles. The carcinogens in tobacco are partially absorbed by the lungs into the blood. Subsequently, they are concentrated into urine by the kidneys via filtration; thus, the epithelial cells of the bladder cancer are exposed to and damaged by the carcinogens, increasing the risk of bladder cancer development. Furthermore, the prolonged effect of smoking carcinogens may lead to cumulative molecular alterations that have adverse effects on the biological and clinical behavior of bladder cancer, promoting growth and motility [27]. In addition, continuous smoking may weaken the immune response to bladder cancer leading to a higher risk of bladder cancer recurrence and mortality [28].

Notably, former smoking was also associated with an increased risk of bladder cancer recurrence and cancer-specific mortality. An explanation may be that the DNA damage caused by tobacco exposure is irreversible and leads to permanent smoking-derived genetic alterations. Vineis *et al* reported that the risk of bladder cancer in former smokers remained higher than the risk in never smokers, even 15 years after quitting cigarette smoking [29]. To assess the irreversible smoking-derived transcriptional changes after smoking cessation, the correlation of the alterations in certain genes (e.g. hypoxia-inducible factor-1) in former smokers with those in current smokers were analyzed. Although the expression changes were weaker in former smokers compared with current smokers, the alterations were still observable in former smokers [30].

Although our study confirms that smoking exposure is significantly related to poorer prognosis in patients with bladder cancer, the study has limitations with regard to investigating the influence of the timing of smoking cessation. On the basis of our findings, former smokers appear to harbour the same risk of recurrence and mortality as current smokers. However, the results require further investigation. Rink *et al* reported that smoking cessation for >10 years may reduce the risk of disease recurrence, cancer-specific mortality and overall mortality [7, 8]. The favourable results may be caused by a decrease in the damaging effects of smoking, improved repair mechanisms, and recovery of defense mechanisms following long-term smoking cessation [9, 31]. Although other studies reported that a longer duration since smoking cessation did not improve disease prognosis, smoking cessation is still advocated due to the well-established beneficial effects on the risk of developing several other diseases, including cardiovascular disease and second primary cancers [10].

There are several potential limitations that should be considered when assessing the results of the present study. First and foremost, there are no prospective studies assessing the impact of smoking status on the outcomes of bladder cancer patients treated with surgery. In the current meta-analysis, 13 retrospective cohorts and 2 case-control studies were included, which were prone to have recall bias. Secondly, substantial heterogeneity was observed among the studies in certain pooled risk estimates, which may have been caused by the variability of smoking exposure, such as in smoking products (cigars, pipes and tobacco chewing), forms of exposure (second-hand smoking and occupational exposure) and the form of surgery (lymph node dissection templates, number of lymph nodes removed, effect of repeat TURB and quality of the TURB). Additionally, we could not adjust for the number and experience of the surgeons and pathologists across the study centers, which may influence the results. Furthermore, the smoking status was predominantly self-reported in the studies and was not verified by biochemical analysis, and is thus subject to recall bias.

In summary, the findings from the present meta-analysis provide evidence that both current smoking and former smoking are associated with a higher risk of recurrence and mortality in patients with bladder cancer. Prospective and larger epidemiological studies with a longer follow-up are required to confirm these findings.

## MATERIALS AND METHODS

### Search strategy

Our meta-analysis was performed according to the meta-analysis of observational studies in epidemiology guidelines [32]. The literature search was retrieved in Pubmed, Embase and the Cochrane Library to identify the eligible studies up to April 2016. The primary search string included the following items: smok* or tobacco or cig*; bladder or urothelial or transitional cell; carcinoma or cancer or neoplas* or tumor; outcome or prognos* or recur* or progress* or mortality or death or survival. The search was focused on human studies, without any other restriction. Furthermore, we also checked relevant review articles and their references to identify all available studies that may not have been included in the primary search results. Additionally, in view of the large number of bladder cancers arising in China, we have also searched the China National knowledge infrastructure (CNKI).

### Inclusion and excluded criteria

Two investigators assessed the eligibility of each study independently. A study would be included if it met the following criteria: (a) published as an original article; (b) patients treated with surgery for bladder cancer; (c) compared with never/no smoking, former smoking/current smoking as the exposure; (d) reported risk estimates (hazard ratios, risk ratios, odd ratios) with corresponding 95% confidence intervals or provided sufficient data to estimate these. Considering there were minor variations in the definitions of smoking status across studies, we defined the smoking status consistent with most of the studies to minimize error and combine the results. The category of “current smoking” comprised the patients who were still smoking at the time of diagnosis or stopped smoking within one year of diagnosis. Patients who had quitted smoking at least one year before the diagnosis of bladder cancer were categorized as “former smoking”. “No/never smoking” was defined as patients who reported to have never smoked in their lifetime. The outcomes of bladder cancer were defined as “disease recurrence”, “disease progression”, and “cancer-specific mortality”, which were synthesized respectively in this meta-analysis.

The excluded criteria were: (a) meeting abstracts, expert opinions or reviews without usable data reported; (b) published as a duplicate article or reported the same population; (c) did not clearly describe smoking exposure categorizations and corresponding effect value. If the studies were reported from the same or overlapping cohort, only the most recent and informative one would be included.

### Data extraction and quality assessment

Two researchers independently extracted the information from eligible studies in a standardized data collection form. The extracted information included the first author, year of publication, study design, database and country, study period, sample size, the length of follow-up, age, sex, diagnosis, surgical method, additional intervention, and the outcome effect values based on smoking status. If a study contained multiple data sets, the data from the main multivariable model, which included more adjusted confounders, was used. For studies in which the effect estimate of the current/former smoking category was potentially acceptable, we attempted to perform analysis with the reported data or contact the author for more detailed information. The quality of the included studies was assessed using the NOS, which consisted of three factors: Patient selection; comparability of combination therapy and targeted therapy alone groups; and assessment of outcome. Studies with higher scores represent higher quality [33].

### Statistical analysis

Smoking status of patients in many studies were usually categorized as current smoking, former smoking and never smoking. To avoid confusion, all statistical analyses in this study were based on comparisons of current/former with never smoking. Statistical heterogeneity among studies was measured using the Q and I^2^ statistic (*p*<0.10 was considered significant statistical heterogeneity). Fixed or random effects models were used to calculate summary relative risk estimates (SRRE), 95% confidence intervals (CI), and corresponding *p-*values for heterogeneity. Forest plots were also applied to assess the relationship between smoking status and outcomes of bladder cancer after surgery.

Subgroup and meta-regression analyses were conducted to examine potential sources of heterogeneity on the basis of study design (single center vs. multicenter), geographic region (America, Europe or Asia), disease stage (muscle-invasive bladder cancer, non-muscle-invasive bladder cancer or urinary bladder cancer) and surgical method (RC vs. TURB). Sensitivity analyses were performed to clarify whether the results were influenced by a particular study, by omitting one study at a time. In addition, to investigate whether publication bias may affect the validity of the estimates, we applied Egger's and Begg's tests to assess the potential bias captured by the funnel plot. All statistical analyses were performed with STATA Statistical Software (version 12.0). P<0.05 was considered to indicate a statistically significant difference, except where specifically noted.

## References

[1] Antoni S, Ferlay J, Soerjomataram I, Znaor A, Jemal A, Bray F (2016). Bladder Cancer Incidence and Mortality: A Global Overview and Recent Trends. Eur Urol.

[2] Li F, Yu Z, Chen P, Lin G, Li T, Hou L, Du Y, Tan W (2016). The increased excretion of urinary orosomucoid 1 as a useful biomarker for bladder cancer. Am J Cancer Res.

[3] Nuhn P, May M, Sun M, Fritsche HM, Brookman-May S, Buchner A, Bolenz C, Moritz R, Herrmann E, Burger M, Tilki D, Trojan L, Perrotte P, Haferkamp A, Hohenfellner M, Wieland WF (2012). External validation of postoperative nomograms for prediction of all-cause mortality, cancer-specific mortality, and recurrence in patients with urothelial carcinoma of the bladder. Eur Urol.

[4] Li F, Hong X, Hou L, Lin F, Chen P, Pang S, Du Y, Huang H, Tan W (2016). A greater number of dissected lymph nodes is associated with more favorable outcomes in bladder cancer treated by radical cystectomy: a meta-analysis. Oncotarget.

[5] Zeegers MPA, Tan FES, Dorant E, Van Den Brandt PA (2000). The impact of characteristics of cigarette smoking on urinary tract cancer risk: A meta-analysis of epidemiologic studies. Cancer.

[6] King B, Dube S, Kaufmann R, Shaw L, Pechacek T (2011). Vital signs: Current cigarette smoking among adults aged ≥18 Years — United States, 2005–2010. Morbidity and Mortality Weekly Report.

[7] Rink M, Furberg H, Zabor EC, Xylinas E, Babjuk M, Pycha A, Lotan Y, Karakiewicz PI, Novara G, Robinson BD, Montorsi F, Chun FK, Scherr DS, Shariat SF (2013). Impact of smoking and smoking cessation on oncologic outcomes in primary non-muscle-invasive bladder cancer. European Urology.

[8] Rink M, Zabor EC, Furberg H, Xylinas E, Ehdaie B, Novara G, Babjuk M, Pycha A, Lotan Y, Trinh Q-D (2013). Impact of smoking and smoking cessation on outcomes in bladder cancer patients treated with radical cystectomy. European urology.

[9] Chen CH, Shun CT, Huang KH, Huang CY, Tsai YC, Yu HJ, Pu YS (2007). Stopping smoking might reduce tumour recurrence in nonmuscle-invasive bladder cancer. BJU International.

[10] Grotenhuis AJ, Ebben CW, Aben KK, Witjes JA, Vrieling A, Vermeulen SH, Kiemeney LA (2015). The effect of smoking and timing of smoking cessation on clinical outcome in non-muscle-invasive bladder cancer. Urologic Oncology.

[11] Serretta V, Altieri V, Morgia G, Di Lallo A, Carrieri G, Allegro R (2013). Cigarette smoking status at diagnosis and recurrence in intermediate-risk non-muscle-invasive bladder carcinoma. Urology.

[12] Lee C, Kim KH, You D, Jeong IG, Hong B, Hong JH, Ahn H, Kim C-S (2012). Smoking and survival after radical cystectomy for bladder cancer. Urology.

[13] Crivelli JJ, Xylinas E, Kluth LA, Rieken M, Rink M, Shariat SF (2014). Effect of smoking on outcomes of urothelial carcinoma: a systematic review of the literature. Eur Urol.

[14] Rieken M, Xylinas E, Kluth L, Crivelli JJ, Chrystal J, Faison T, Lotan Y, Karakiewicz PI, Sun M, Fajkovic H, Babjuk M, Bachmann A, Scherr DS, Shariat SF (2014). Effect of diabetes mellitus and metformin use on oncologic outcomes of patients treated with radical cystectomy for urothelial carcinoma. Urologic oncology.

[15] Rink M, Xylinas E, Trinh QD, Lotan Y, Margulis V, Raman JD, Fisch M, Lee RK, Chun FK, Abdennabi J, Seitz C, Pycha A, Zlotta AR, Karakiewicz PI, Babjuk M, Scherr DS (2013). Gender-specific effect of smoking on upper tract urothelial carcinoma outcomes. BJU International.

[16] Da Silva RD, Xylinas E, Kluth L, Crivelli JJ, Chrystal J, Chade D, Guglielmetti GB, Pycha A, Lotan Y, Karakiewicz PI, Sun M, Fajkovic H, Zerbib M, Scherr DS, Shariat SF (2013). Impact of statin use on oncologic outcomes in patients with urothelial carcinoma of the bladder treated with radical cystectomy. Journal of Urology.

[17] Kim PH, Kent M, Zhao P, Sfakianos JP, Bajorin DF, Bochner BH, Dalbagni G (2014). The impact of smoking on pathologic response to neoadjuvant cisplatin-based chemotherapy in patients with muscle-invasive bladder cancer. World Journal of Urology.

[18] Sfakianos JP, Shariat SF, Favaretto RL, Rioja J, Herr HW (2011). Impact of smoking on outcomes after intravesical bacillus Calmette-Guerin therapy for urothelial carcinoma not invading muscle of the bladder. BJU international.

[19] Chade DC, Shariat SF, Godoy G, Savage CJ, Cronin AM, Bochner BH, Donat SM, Herr HW, Dalbagni G (2010). Clinical Outcomes of Primary Bladder Carcinoma In Situ in a Contemporary Series. Journal of Urology.

[20] Leibovici D, Grossman HB, Dinney CP, Millikan RE, Lerner S, Wang Y, Gu J, Dong Q, Wu X (2005). Polymorphisms in inflammation genes and bladder cancer: From initiation to recurrence, progression, and survival. Journal of Clinical Oncology.

[21] Pastore AL, Palleschi G, Fuschi A, Silvestri L, Al Salhi Y, Costantini E, Zucchi A, Petrozza V, de Nunzio C, Carbone A (2015). Can daily intake of aspirin and/or statins influence the behavior of non-muscle invasive bladder cancer? A retrospective study on a cohort of patients undergoing transurethral bladder resection. BMC Cancer.

[22] Allard P, Fradet Y, Têtu B, Bernard P (1995). Tumor-associated antigens as prognostic factors for recurrence in 382 patients with primary transitional cell carcinoma of the bladder. Clinical Cancer Research.

[23] Ahirwar D, Kesarwani P, Manchanda PK, Mandhani A, Mittal RD (2008). Anti- and proinflammatory cytokine gene polymorphism and genetic predisposition: association with smoking, tumor stage and grade, and bacillus Calmette-Guerin immunotherapy in bladder cancer. Cancer genetics and cytogenetics.

[24] Wyszynski A, Tanyos SA, Rees JR, Marsit CJ, Kelsey KT, Schned AR, Pendleton EM, Celaya MO, Zens MS, Karagas MR (2014). Body mass and smoking are modifiable risk factors for recurrent bladder cancer. Cancer.

[25] Stern MC, Lin J, Figueroa JD, Kelsey KT, Kiltie AE, Yuan JM, Matullo G, Fletcher T, Benhamou S, Taylor JA, Placidi D, Zhang ZF, Steineck G, Rothman N, Kogevinas M, Silverman D (2009). Polymorphisms in DNA repair genes, smoking, and bladder cancer risk: findings from the international consortium of bladder cancer. Cancer research.

[26] Zeegers MP, Goldbohm RA, van den Brandt PA (2002). A prospective study on active and environmental tobacco smoking and bladder cancer risk (The Netherlands). Cancer Causes & Control.

[27] Reznikoff CA, Sarkar S, Julicher KP, Burger MS, Puthenveettil JA, Jarrard DF, Newton MA (2000). Genetic alterations and biological pathways in human bladder cancer pathogenesis. Urologic oncology.

[28] Sopori M (2002). Effects of cigarette smoke on the immune system. Nature Reviews Immunology.

[29] Vineis P, Esteve J, Hartge P, Hoover R, Silverman DT, Terracini B (1988). Effects of timing and type of tobacco in cigarette-induced bladder cancer. Cancer research.

[30] Gabriel U, Li L, Bolenz C, Steidler A, Kränzlin B, Saile M, Gretz N, Trojan L, Michel MS (2012). New insights into the influence of cigarette smoking on urothelial carcinogenesis: Smoking-induced gene expression in tumor-free urothelium might discriminate muscle-invasive from nonmuscle-invasive urothelial bladder cancer. Molecular carcinogenesis.

[31] Strope SA, Montie JE (2008). The causal role of cigarette smoking in bladder cancer initiation and progression, and the role of urologists in smoking cessation. The Journal of urology.

[32] Stroup DF, Berlin JA, Morton SC, Olkin I, Williamson GD, Rennie D, Moher D, Becker BJ, Sipe TA, Thacker SB (2000). Meta-analysis of observational studies in epidemiology: a proposal for reporting. Jama.

[33] Stang A (2010). Critical evaluation of the Newcastle-Ottawa scale for the assessment of the quality of nonrandomized studies in meta-analyses. European journal of epidemiology.

